# Book Review

**DOI:** 10.1120/jacmp.v4i4.2508

**Published:** 2003-09-01

**Authors:** Walter H. Grant III

**Affiliations:** ^1^ Associate Professor of Radiology Baylor College of Medicine One Baylor Plaza Houston TX 77030

## Abstract

**A Practical Guide to Intensity Modulated Radiation Therapy**. Memorial Sloan‐Kettering Cancer Center (MSKCC) Staff, Medical Physics Publishing, Madison, WI, 2003. 450 pp. Price: $125.00. ISBN: 1‐30524‐13‐7.

PACS number(s): 87.53.‐j, 87.53.Xd, 87.53.Kn

It is challenging to review such a wealth of information and create a synopsis that tries to be fair as well as accurate. This book contains 19 chapters (over 400 pages) of information on IMRT Although the title identifies it as a “Practical Guide” and Dr. Samuel Hellman, in the Foreword, describes it as a “User's Guide,” understandably, there is a distinct emphasis on IMRT as practiced at the author's institution. If you consider the leading efforts of MSKCC in IMRT, any physicist or physician who is serious about delivering IMRT will want this book in his/her library.

Although the book contains 19 chapters, I subdivided the book into three sections. The first section is eight chapters of “Introductory IMRT” and comprises about 50% of the book. It is more physics driven and basic, thereby serving as a reference for someone who wants to begin IMRT. These chapters contain definitions of IMRT terminology as well as the history for the ideology that has developed at MSKCC.

The second section involves six chapters on the clinical applications of IMRT. The disease sites covered are prostate cancer, head and neck cancer, pediatric cancers, breast cancer, non‐small cell lung cancer, and cancers requiring large treatment fields. These chapters are filled with references to the works of others and the rationale for treatments at MSKCC. Most informative are the fine details regarding anatomy delineation that are frequently hard to extract from papers or talks as well as an honesty about the current status of unresolved issues. In addition the chapters contain excellent, succinct summaries.

The last five chapters cover IMRT as an advancing technology. There are chapters on biological models, geometric uncertainty considerations, radiation protection, and advanced planning techniques. As these are current issues, they serve more as stimuli for thought rather than a finished product. I spent more time per chapter here than elsewhere.

What will you not find in the book? Other ways and philosophies for doing IMRT! For example, you will find the chapters on optimization and treatment planning marginally useful if you do not use the MSKCC planning system, and you don't. While there are some generic discussions of multileaf collimators from all the vendors, the illustrative examples involve only the equipment at MSKCC. The same is true for the portal imaging system. I thought the book could have used a chapter on immobilization systems and the tricks associated with them.

Since IMRT was a major undertaking at this massive institution, many different authors write the chapters. In three chapters I had to read about how to handle the tongue and groove effect of the MLC. In at least seven chapters the phrase “dose painting” was used, but in only one chapter was it stated that “dose painting” would not be implemented until technical issues were resolved. I suspect in later editions this type of disjointed compilation of writings will be smoothed and while annoying, it by no means lessens the value of this work as a reference book. In fact, as a ten‐year veteran of IMRT with only tomotherapy, I believe I will use this book frequently over the next few years and thank the authors for placing all this information in one book.

